# Supplementary material for: Patterns of Intron Gain and Loss in Fungi
Source: PLoS Biol. 2004 Nov 30;2(12):e422. doi: 10.1371/journal.pbio.0020422 (PMC532390; doi:10.1371/journal.pbio.0020422)
Supplement: Table S1 — Also available at http://genes.mit.edu/NielsenEtAl/. (4.3 MB ZIP). [file pbio.0020422.st001.zip › NielsenEtAl/html/1340.html]

AN6685.1.NCU03045.1.MG04703.1.FG07053.1


```
 CLUSTAL W (1.82) Multiple Sequence Alignments - Introns Inserted


Sequence 1: NCU03045.1	1252 aa
Sequence 2: MG04703.1	1219 aa
Sequence 3: FG07053.1	1262 aa
Sequence 4: AN6685.1	1214 aa
Alignment Length: 1303 aa
Number Identitical Residues: 325 aa
Alignment Score (without introns) 21510


MG04703.1 	-------~--------------------------MPDS------------FSSLLT--LC
NCU03045.1	-------~------------------------MRLPFSRRSDARGQSLSPISVLLT--LS
FG07053.1 	MLRYRIA0IWLYCAATHVSDIDYIDSKRKLTVMQLSLQRRGPSARQRHGLGPVLGSSLVA
AN6685.1  	-------~------------------------MRLPSLFGPATAG-----SSAFLQ--LW
          	                                  :.      :        . :    : 

MG04703.1 	ALASIPTANAFDFKPAPEANLDLSQLGRVGLAGDFSGLSLYQYEGQSESQFNANGSESLL
NCU03045.1	SVALSPVT-AISFTPAPATNLDLKDLGRVALAGDFSGISLFQFEEQNEQPFTTNGSQSLM
FG07053.1 	LAALAPFTQAITFNSVPSPNLDFSNLGRIGIVGDFDGISLYEYEGQNESPRNSNGSESLL
AN6685.1  	LLSQLSGASALSFQPVSRPELDLSRLGHVALTGEFDAVTFYSYAEQKNAKAGDDDSQSIL
          	  :  . :.*: * ... .:**:. **::.:.*:*..::::.:  *.:     :.*:*::

MG04703.1 	ARAPNGALATVVGTDASINAMCAYVLSNGDLAGVVLGGNFTSIGT----------QKSPA
NCU03045.1	ARMPNGIFSPVVTTDATILAMCTFVEGNGKEDGVIIGGNFTSLQLPTHGSGSPDIRESKA
FG07053.1 	AQLPNGAFTSILSSDASIRAMCSFQLSNGEMQGVVIGGNFTSLDG----------TQSKA
AN6685.1  	APLPNGILTTLSTSDADIRAMCAFSKQDGSHSGIYVGGNFTSLGG----------EKANG
          	*  *** ::.:  :** * ***::   :*.  *: :******:             :: .

MG04703.1 	IAIFNPNTSEATPLPGLEGRVDAVLCDQESSTIYVGGTFRGNSSTNAITWKNNQ-WTDLP
NCU03045.1	IALFNPNTSSIETLSGLEGQVYAVLCDRDTNSVYVGGNFVGKDSRNAIAWVGGQGWTDLP
FG07053.1 	VALFNPNTTKITPLDGIEGEVNAVYCDQERDAVYVGGNFKGANSTNAIAWVGTDGWTNLP
AN6685.1  	VALYDPISNKVTTLPGLSGSVSALLCDQETNTVYVGGSFKQGNTSNAAAWVVGEGWKDLS
          	:*:::* :..  .* *:.* * *: **:: .::****.*   .: ** :*   :.*.:*.

MG04703.1 	FGGFNGQVRSISKASNGHIIFGGEFTGLGNGSLPSHPDGQAINIDKSTITAGSSSSTNGF
NCU03045.1	FRGFNGPVNSITKASNGHIIYGGSFTGLGNATAPTERDGQVINLSSANISASPSTTRDGF
FG07053.1 	FAGFNGPVKAITKASNGHIIFGGTFTGLGNSSAPNESISQVINLSTANITDDNGSGTD--
AN6685.1  	FGGLNGPVSSIVKNDDAHIVFGGSFDGIGNSTSSSKKIEQVINLSNATITSDTTSTRSGF
          	* *:** * :* * .:.**::** * *:**.: ...   *.**:..:.*: .  :  .. 

MG04703.1 	SDPRNIVCKAPGATGAGNTWLLQDNTPGFWQASFKFGFQPTKLRLRNTHQDGRGTRTWRF
NCU03045.1	SDPKNIVCSTSGTDGAGKTWLLQDNSPGFWQAKFRHGFRPTKLRLYNTHLDGAGTKTWRF
FG07053.1 	--PQDIVCPGSSSS----PWLLQDSTPGYWEAAFSFGFEPTKLRLKNTRQDGRGTKTFRF
AN6685.1  	TDPRNIICQTSGAGGAGQTWLLADYALGYWRADMRYNFSPTKLRVYNTHYEGRGTKSFLF
          	:.*::*:*  ..: .:...*** * : *:*.* : ..* *****: **: :* **::: *

MG04703.1 	TAQPINGIMNFTYIDPATGRNASCTSECPLSDDTNLPFQDFHFVNVIGMNGFRIDISQFY
NCU03045.1	TALPINGIMNMTYVDPVTNKNATCTSECPLSNNQTIKFQDFHFINVIGMNEFRIDISDYY
FG07053.1 	VAYPIDGIMNLTYVDPESGNNVTCTSECPLLDND--EYQDFTFVNRVGMNRFRLAVSEWY
AN6685.1  	RRLPDNGIMNLTYTDPSTGDDVNCDQYCSLSDDPDEEYRDFRFVNQVGMTGFMFEIRDYY
          	   * :****:** ** :. :..* . *.* ::    ::** *:* :**. * : : ::*

MG04703.1 	GSGGGLDGIELFQDD~IFTYAINDFNEPSCAGATKPASVTATGPFSVSASLQSQSQYLSA
NCU03045.1	GNGGGLNGIELFGDD~IFSYAINDFNEPTCANLEFKSSSTATGPWQVTPSHQSTSEYLTA
FG07053.1 	GNGGGLEAIELYQSD~IYAYAISDFNEPKCGGVKNPSSATHTGPWTVSPSLESSSEYLVT
AN6685.1  	GTGAGLNGIEVFTND1VFTYAINDFNEPTCGNSSDISTSTRTGSWTLTDGNDSPSDYLTA
          	*.*.**:.**:: .* :::***.*****.*..    :: * **.: :: . :* *:** :

MG04703.1 	RIASPVTSDSASVVFSPDIRSSGDFSVNMYTPGCIPDNTCDTRGQVNVT----INPRPGV
NCU03045.1	QISPPVTDTSAKIVFSPDIRESGFYSVNLYTPGCLQDDTCSSRGQVHLSGQMTADPTKSE
FG07053.1 	ELNNDYDPESISVTFYPNIMESGNYSVNIYTPGCRADDTCDSRGRVNITGIMSGDDGKDT
AN6685.1  	HVS-DSDAASTSVVFEPNIQRSGNYSILVYTPGCVQDGTCGSRGIVNVTATVRADSE--E
          	.:       * .:.* *:*  ** :*: :*****  *.**.:** *:::.    :     

MG04703.1 	TQSVQLFQTNNFEKYDQIFFGQMQTTG-SFRPTITMTPLNGQSLDTMTFVAQRVGFVQVN
NCU03045.1	PVDQTLFQTNNFDKYDQIYNGMVDASSSSFRPQITLTPVAGQDLASLTLVAQSIAFTLIN
FG07053.1 	NFTSQLFQTNYFDKYDQIYFGYIEKSSASFKPSVTLRPVGGQEIDPLTIVAQRVEFTLTN
AN6685.1  	LIEKQIYQTNNYEKYDTIYTGPVDVSDDSFRPRVTLSPVAGQ--GDLTVVASRVRFELIS
          	     ::*** ::*** *: * :: :. **:* :*: *: **    :*.**. : *   .

MG04703.1 	ST----GGLNGLFDWNPTKTTIDANEFESSPFHKLGSSLGPSTTVVALAAAGDVTFVGGS
NCU03045.1	ST----GGLNGLFEYTPG-QHVNVSDFTASAVNRLGSSFSGKSAVNTLATSGDTVFVGGN
FG07053.1 	ST----GGLNGLFDFDPEQAVVNTSSLTNSPINKLGASFDRSSGVSVLVTDSNTTYVGGN
AN6685.1  	ASNDTSGELNGLFEYDPTSKNATANLLG-STINSSGTQLDSDASVLSLVTHRGVIYAGGN
          	::..::* *****:: * .    .. :  *..:  *:.:. .: *  *.:  .. :.**.

MG04703.1 	FKGDGAQNIVGVSSGNNAGAQSLSGGLNGPVATMALTGTKLYVGGSFSATQDNSVNNLDN
NCU03045.1	FTSPSAEHVVALNTKDST-NRTLDGGLNGEVQSMILDGNTLYVGGSFNSTSEKPVDGLNN
FG07053.1 	FTSKDHDNILAIIENDDV--KEPGGGLNGEVFAMYLNDTQLYVGGDFSNTDNGPVTGLDN
AN6685.1  	FSSSDINNLFSLEQDANA-TALPQGGLNSEVTTMSVLNDKLYVGGNFTGTSEGATDSLSY
          	*.. . :::..:    ..      ****. * :* : .  *****.*. *.: .. .*. 

MG04703.1 	VAVLDTATRTWTALGGGVEGNVRNAVLMTMNITEASDETVVAFSGSFRQLRSFDGNPAVP
NCU03045.1	VAAYDTEKNTWSPLGAGVNGKVMRVVGLTMNITGSTPEFVVSVNGDFDELNAFDKNPAVS
FG07053.1 	VAVYDTKEDKWSPLGAGVDGPVKYAVPLMVNITADTPELVIAFTGSFSSCNAFDDYSSIS
AN6685.1  	VASYSIDSKSWSALGGGVNGHVKRVFALPLNVSTVINETIVAVSGDFDELLPFDEYPAVA
          	**  .    .*:.**.**:* *  .. : :*::    * :::..*.* .  .**  .::.

MG04703.1 	VNGFAVWVPSKRNWLNNLD-GVEAIDGLLSSSILDVPGDG-NFYAGTVSSASLSAHGAAT
NCU03045.1	VTGFGIWVPSQKNWLQNLDLELESIDGVLSTSIIDRSGSG-SLYAGSLVSSTLGVRDIAG
FG07053.1 	VDGFAIWVASKGNWLQNLDSNYPAFGGSLTAAVLDIPMDGGSLYAGALSSAQLGAGGAAT
AN6685.1  	VSGFAVWVPSRNNWLQNLNVSQTQFAGHLSS---FASFNGTTILGGSLSSGGLAAGGAVA
          	* **.:**.*: ***:**:     : * *::     . .* .: .*:: *. *.. . . 

MG04703.1 	LFGNSVGRFPAKIQSSAASSSTSNLSKRDSSLNSGSS----GVNAGAFYGNDGRNMTVLA
NCU03045.1	-LGETLSRLPLKIQAPSKPSSSNSLAKRDSSINADSADSLQGVVTGIFDQENKRNLTILG
FG07053.1 	LSDEGLGQFPLNIRTSTSDSKSSSLSRRDVTSFDKNT---TGVVTGIFYDDNDYNITVLA
AN6685.1  	LLQEDGLQLGPLLNSTQLTG---------------------EIITGIYDTDSSRNLTILG
          	   :   ::   :.:.   .                      : :* :  :.  *:*:*.

MG04703.1 	GHFTATATDGNTVHNIVIVDGGDKDTVTGLSQ-LSDNSTFRAVAVQGDTLFAGGNVTGNV
NCU03045.1	GHFTATGSE-PAINNLLIMDGKKNNEISGLGDGISQDSTVMALVVHNDILFAGGNITGTV
FG07053.1 	GHFTTESTKGEKIQNIALLED---DTVTGLGSDISNSSTFVALAVRDGILFAGGDISGTI
AN6685.1  	GHFN-LSTGGQEVRNLAFIDGG-TGSISGLGTEVENNSTFMAFAITSNVLYAGGRVTGTV
          	***.  .: .  :.*: :::.  .. ::**.  :.:.**. *..: .. *:*** ::*.:

MG04703.1 	NGVTVSGLISYNLASKTYNT-QPPALSGGNSTVQTIVARPE-SSDVYVGGSFTLAGSLGC
NCU03045.1	NGNEVKALVTYNLASMSFSS-QPPSLNGGDGTVSSIAVRDG-TDDVYVGGSFTQAGSLDC
FG07053.1 	DGTDIRGLVSYNLESKSFNS-QPASVSGANGTVAAIAVRPGDSGDVYVGGSFETAGALDC
AN6685.1  	GKNEIDGLVLYDLQNGTIAQEQPSKFNGDNVSVNAIAPRPN-SKEIYVGGYFQAAGALTC
          	.   : .*: *:* . :   .**. ..* : :* :*. *   : ::**** *  **:* *

MG04703.1 	PGVCYYSASSGVWNRPGSNLEGSVHSLLWTSNSVLVAGGNMTVNDTIKTNLARYNTGSSS
NCU03045.1	PGVCFFSTSSSQWQQAGQNLGGTVNTLLWVDENLLLAGGNLTINNTASAYLATYDPKTLV
FG07053.1 	PGLCTYNVNSGQWNRPGSQLEGTVFSLLWMSKNQLVVGGDLRGNGSSQRYLSSWDANAES
AN6685.1  	PGVCYYDTESQQWNRPGVGLEGSVLALKWGNANTLIAVGNLTVAGN-ETAVASYSAKEQI
          	**:* :...*  *::.*  * *:* :* * . . *:. *::   .. .  :: :..    

MG04703.1 	WDAFPGADQ--LPGPVEVLTPASRDGDQIWASGTSTTNGLVYIMKY---DGSKWIEA-PP
NCU03045.1	WDAYPDAGQ--LPGPVEVLTAGTSDNKQIWVSGTAS-NGSVYLMKSEKDDGSSWHSVGQT
FG07053.1 	WSNFPGSED--IPGPISVISPGSSDYQQLWVAGTSSKDDSVFVMKY---DGEKWHTVKPS
AN6685.1  	WEVFDGASSSSIPGTVTAFSPANGDASRFWLAGQSS-NGSNFIVNY---DGSSFQLAQEI
          	*. : .: .:::**.: .::... * .::* :* :: :.  ::::    **..:  .   

MG04703.1 	LGPGSVVRSLQVFTLTSDHDGTDIVLSNQALMLTGSIRIPNFGSASAALFDGTSYQAYAL
NCU03045.1	LQPGTKIRGLQIFQLSQSHDKHSLIDDKEVLMVTGHIVLPDFGSASGVLFDGANFRPYIL
FG07053.1 	LPSDTIIRGLQVFSVTEDHGDADLLDKDQVLMLTGSIDIPDFGVASAALFNGTAYQPYAM
AN6685.1  	FDDGTIIRGLDVIPLASDHDDSDLLNNDLALMITGQIAVPEFGTASAALFNGTTLIPFVI
          	:  .: :*.*::: ::..*.  .:: .. .**:** * :*:** **..**:*:   .: :

MG04703.1 	TTSSDRTPGSISRVFVEKSQFFTSSE1G~GRLPLVAIVLIGLGISLVLMLLIVLAGLFMD
NCU03045.1	TTNSGNTPGSLAVVFSEKQNFFGK-D~G~KHLPLVFVVLIGLGISLALMLLIVVAGLILD
FG07053.1 	TIKAGDGPGTIASIFSQRDSFFKE--~E1KHMALGFVVLIGLAIALGLMLLLVLCGIILD
AN6685.1  	SSKSDGQPGRMSRMFYENNNPYAR-G~S1GHHSNGIVVLVAFCCALGCVFLIVIAGVIFN
          	: .:.  ** :: :* :... :        : .   :**:.:  :*  ::*:*:.*::::

MG04703.1 	RLRKKREGYVPAPTSM-YDRGSGIQRIPPGELLSSLNQGRPGAPTI-
NCU03045.1	RIRKKREGYVPAPTHM-YDRGSGIQRIPPHELLEGLSKSRSGAPQM-
FG07053.1 	RIRKKREGYVPAPTSM-YDRGSGMRHIPPHELLESLGHGRGGAPPRV
AN6685.1  	KIQRRRQGYMAAPQSTGTDRPTSMRRLPPEYLFNSLKQPNPGAPSI-
          	:::::*:**:.**   . ** :.::::**  *:..* : . ***
```
